# Discriminating the Short-Term Action of Root and Foliar Application of Humic Acids on Plant Growth: Emerging Role of Jasmonic Acid

**DOI:** 10.3389/fpls.2020.00493

**Published:** 2020-04-28

**Authors:** David De Hita, Marta Fuentes, Victoria Fernández, Angel M. Zamarreño, Maite Olaetxea, Jose M. García-Mina

**Affiliations:** ^1^Department of Environmental Biology, Biological and Agricultural Chemistry Group (BACh), University of Navarra, Pamplona, Spain; ^2^Forest Genetics and Ecophysiology Research Group, School of Forest Engineering, Technical University of Madrid, Madrid, Spain; ^3^Centre Mondial de I’lnnovation Roullier, Saint-Malo, France

**Keywords:** humic substances, humic acids, foliar application, root application, shoot growth, root growth, jasmonic acid, salicylic acid

## Abstract

Humic substances (HS, fulvic and humic acids) are widely used as fertilizers or plant growth stimulants, although their mechanism of action still remains partially unknown. Humic substances may be applied either directly to the soil or as foliar sprays. Despite both kind of application are commonly used in agricultural practices, most of the studies regarding the elicited response in plants induced by HS are based on the root-application of these substances. The present work aimed at discriminating between the mechanisms of action of foliar application versus root application of a sedimentary humic acid (SHA) on plant development. For this purpose, six markers related to plant phenotype, plant morphology, hormonal balance and root-plasma membrane H^+^-ATPase were selected. Both application strategies improved the shoot and root growth. Foliar applied- and root applied-SHA shared the capacity to increase the concentration of indole-3-acetic acid in roots and cytokinins in shoots. However, foliar application did not lead to short-term increases in either abscisic acid root-concentration or root-plasma membrane H^+^-ATPase activity which are, however, two crucial effects triggered by SHA root-application. Both application modes increased the root concentrations of jasmonic acid and jasmonoyl-isoleucine. These hormonal changes caused by foliar application could be a stress-related symptom and connected to the loss of leaves trichomes and the diminution of chloroplasts size seen by scanning electron microscopy. These results support the hypothesis that the beneficial effects of SHA applied to roots or leaves may result from plant adaptation to a mild transient stress caused by SHA application.

## Introduction

There is a growing interest in the development and implementation of more sustainable land management practices, aiming to stop the progressive degradation of soils while maintaining or enhancing food production in a context of increasing demands. Among the different strategies, the use of humic-based soil amendments constitutes an environmentally friendly approach. Many studies have shown that humic substances (HS) from different origins applied to plant roots can improve plant growth and mineral nutrition (see reviews by Chen et al., 2004; Rose et al., 2014; Olaetxea et al., 2018; and references therein). These positive effects involve various mechanisms, including the action of HS on soil and rhizosphere properties, as well as their interactions with plant roots.

The capacity of HS to enhance plant growth has promoted the development of humic-based commercial products for plant production (Rose et al., 2014; Canellas et al., 2015; Olk et al., 2018). In general, commercial HS-based products can be applied not only to the soil (root area) but also as foliar sprays (Rose et al., 2014; Canellas et al., 2015). While the mechanisms of action involved in the plant growth promoting effect of soil-applied HS have been the subject of different studies (Pinton et al., 1999; Nardi et al., 2002; Chen et al., 2004; Berbara and García, 2013; Canellas et al., 2015; García et al., 2016b; Olaetxea et al., 2018), the beneficial action of foliar-applied HS remains unexplored to date. Indeed, it is assumed that foliar-applied HS promote plant growth by mechanisms similar to those involved in HS root application (Rose et al., 2014). However, there are many differences regarding the modes of absorption, transport and interaction of root- versus foliar-applied HS. For example, the range of concentration of HS that is needed to improve plant growth via foliar application is much lower compared to that for root HS application (Chen and Aviad, 1990). Likewise, HS applied to the leaves do not interact with the soil and rhizosphere, where important reactions and interactions that lead to an enhanced nutrient bioavailability take place (Baigorri et al., 2013; Urrutia et al., 2014; Olaetxea et al., 2018; Zanin et al., 2019). It is therefore plausible that the mechanisms underlying the response of plants to foliar-applied HS may involve nutritional, metabolic and physiological differences compared to the response to root-applied HS.

Hence, the aim of this study is to evaluate some of the mechanisms triggered after foliar application of a well-characterized sedimentary humic acid (SHA) previously found to improve plant growth when applied to roots (Aguirre et al., 2009; Mora et al., 2010, 2012, 2014; Olaetxea et al., 2015, 2019). Our hypothesis is that the interaction of HS with plant leaves might induce some kind of mild stress signals that may activate hormonal and molecular pathways involved in the regulation of plant stress responses. As the nature of HS-leaf interactions in the phyllosphere may be quite different from that of HS-root/rhizosphere interactions, we hypothesize the occurrence of potentially different mechanisms responsible for the beneficial effects of both HS supply modes on plant growth.

## Materials and Methods

### Extraction and Purification of a Leonardite HA (SHA)

Sedimentary humic acids (SHA) were obtained from a leonardite originated in the Danube basin (Czechia). The extraction and purification of SHA were performed according to the International Humic Substances Society methodology with some modifications, following the protocol described in detail in Aguirre et al. (2009; Supplementary Information). The main physico-chemical features of SHA are described in Supplementary Table S1 and Supplementary Figures S1, S2.

### Plant Growth and Experimental Design

Cucumber (*Cucumis sativus* L. var. Ashley) seeds were germinated in the dark, on perlite and filter paper moistened with a 1 mM CaSO_4_ solution. The germination chamber conditions were 25°C and 75% relative humidity (RH). One week after, seedlings were transferred to a hydroponic system with vessels filled with 7 L of nutrient solution. This solution contained: 0.63 mM K_2_SO_4_, 0.5 mM KH_2_PO_4_, 0.5 mM Ca(NO_3_)_2_, 0.30 mM MgSO_4_, 0.25 mM KNO_3_, 0.05 mM KCl, 0.87 mM Mg(NO_3_)_2_, 40 μM H_3_BO_3_, 27.3 μM MnSO_4_, 2 μM CuSO_4_, 2 μM ZnSO_4_, and 1.4 μM Na_2_MoO_4_. The solution was supplemented with 80 μM iron as Fe-ethylenediamine-N,N′-bis(2-hydroxyphenylacetic acid) chelate (80% [w/w] ortho-ortho-isomer). The average value of the pH of the nutrient solution during the experiment was 6.7. The different experiments were performed in a growth chamber where the experimental conditions were set up to 25°C/21°C and 70%/75% RH in a day-night cycle and the photoperiod was 15 h/9 h (PAR of 250 μmol m^–2^s^–1^).

In order to assess the effects caused by the foliar application of SHA, several solutions with different SHA concentrations (in the range 20–100 mg C L^–1^), at pH 6, were prepared by dissolving the required amount of SHA in water, with the addition of 0.1% Tween20 (vol/vol). The corresponding treatments were sprayed on both abaxial and adaxial sides of leaves of cucumber plants 10 days after transplantation. Leaves of control plants were treated with 0.1% Tween20 in water (vol/vol). All foliar treatments were always applied 2 h after the start of the diurnal period. Plants were always harvested at the same time of the day (6 h after the start of the light period) to avoid diurnal variations.

An additional experiment was performed in order to explore the effects caused by root-applied SHA on the concentration of jasmonic acid (JA), jasmonoyl-isoleucine (JAIle), and salicylic acid (SA) in plant tissues. In this experiment, plans were grown in the same conditions as described above, and 10 days old cucumber plants were treated with 100 mg C L^–1^ of SHA added to the nutrient solution (SHA.R100).

### Measurement of Root and Shoot Dry Matter

Shoots and roots were sectioned with a scalpel and separated before fresh weight (FW) measurement. Five plants were harvested for each treatment and each harvest time. Root and shoot samples were then dried at 50°C for 3 days in a lab stove, and their dry weight (DW) was subsequently measured individually.

### Mineral Nutrition Analysis

Dried samples (five shoots and five roots for each treatment and harvest time) were used to determine the concentration of the mineral nutrients in leaves. Leaf-samples (0.15 g dry sample) were subjected to acidic digestion (8 mL of 65% HNO_3_ and 2 mL of 33% H_2_O_2_) in a microwave at a controlled temperature of 200°C. Digested samples were then diluted with dH_2_O in 25 mL volumetric flasks, and the nutrient concentrations were measured by ICP-OES (iCAP 7400 DUO, Thermo Scientific).

### Root Morphology

Root morphology images were acquired with the software WINRHIZO (Regent Instruments Inc., Canada) implemented in a scanner (EPSON Perfection V700 Photo). In this study, three plants per treatment and harvest time were analyzed.

### Leaf Morphology

Morphological features of leaves were analyzed by transmission (TEM) and scanning (SEM) electron microscopy. Second true leaves (fully expanded) were harvested after 7 days from the onset of the treatments. For both SEM and TEM, 4 mm^2^ pieces were cut and subsequently fixed in 2.5% glutaraldehyde-4% paraformaldehyde for 6 h at 4°C. Then they were rinsed in ice-cold phosphate buffer, pH 7.2, 4 times within a period of 6 h and left overnight.

For SEM, fixed leaf tissues were dehydrated in a series of absolute ethanol (i.e., 30, 50, 70, 80, 90 and 100%; ×3 times each concentration). They were subsequently subjected to critical point drying (Leica EM CPD300). Before observation, samples were gold-sputter and examined with a JEOL 6400 SEM.

For TEM, fixed and phosphate buffer rinsed cucumber leaf samples were post-fixed for 1.5 h in 1:1 water: 2% aqueous osmium tetroxide solution containing 3% potassium ferrocyanide. Tissue were consequently washed with distilled water (×3), dehydrated in a series of 30, 50, 70, 80, 90, 95, and 100% acetone (×2, 15 min each concentration) and embedded in acetone-Spurr’s resin mixtures (3:1, 2 h; 1:1, 2 h; 1:3, 3 h) and kept in pure resin overnight (kept at 25°C). Pure resin sample embedding was carried out in blocks which were incubated at 70°C for 3 days. Semi-thin leaf sections were cut, mounted on nickel grids and post-stained with Reynolds lead citrate for 5 min, prior to TEM observation (Jeol 1010, equipped with a CCD megaview camera) at 80 kV.

### Determination of Hormones in Roots and Shoots

Cucumber plants (five replicates per treatment and harvest time, with one plant per replicate) were harvested and separated into root and shoot prior to freezing in liquid nitrogen. Samples were reduced to a powder in a Freezer/Mill cryogenic grinder (SPEX SamplePrep) and stored at −80°C prior to analyses.

The content of indole-3-acetic acid (IAA), abscisic acid (ABA), SA, JA, and JA-Ile in plant tissues was analyzed by high-performance liquid chromatography-electrospray-high-resolution accurate mass spectrometry (HPLC-ESI-HRMS). These hormones were extracted and purified as described in Silva-Navas et al. (2019) from 0.25 g of ground frozen plant tissue, homogenized with 2.5 mL of precooled (−20°C) methanol:water:HCOOH (90:9:1, v/v/v, with 2.5 mM Na-diethyldithiocarbamate) and 25 μL of a stock solution of 1000 ng ml^–1^ of deuterium-labeled internal standards in methanol. Samples were shaked in a Multi Reax shaker at room temperature for 60 min at 2000 rpm. Immediately afterward, solids were separated by centrifugation at 20.000 × *g* for 10 min, and re-extracted with 1.25 mL of fresh extraction mixture by shaking for 20 min and subsequent centrifugation. Aliquots of 2 mL of the pooled supernatants were separated and evaporated in a RapidVap Evaporator operating at 40°C. The residue was re-dissolved in 500 μL of methanol/0.133% acetic acid (40:60, v/v) and centrifuged at 20.000 × *g* for 10 min before the injection in the HPLC-ESI-HRMS system. Detailed description of the quantification is reported in Silva-Navas et al. (2019).

The endogenous content of the following cytokinins was also analyzed: *trans*- and *cis*-zeatin (tZ and cZ), dihydrozeatin (DHZ), *trans*- and *cis*-zeatin riboside (tZR and cZR), dihydrozeatin riboside (DHZR), isopentenyladenine (iP), and isopentenyladenosine (iPR). Extraction process was carried out following the method described in Silva-Navas et al. (2019), using 0.25 g of frozen plant material previously ground with liquid nitrogen. Sample homogenization was made with 4 mL of precooled (−20°C) methanol-water-formic acid (15:4:1, v/v/v), and with 25 μL of a stock solution of 100 ng/mL of each deuterium-labeled standard (in methanol). An overnight extraction at −20°C was carried out, after which solids were separated (20.000 g, 10 min, 4°C). Then, they were re-extracted with 2 mL of extraction mixture and centrifuged again. Supernatants were passed through a Sep-Pak C18 cartridge preconditioned with 2 mL of methanol and 2 mL of extraction medium. Afterward, the eluted was evaporated near to dryness with a RapidVap Evaporator and the residue was re-dissolved in 2 mL of 1 M formic acid. This solution was applied to an Oasis MCX column preconditioned with 2 mL of methanol and 2 mL of 1M formic acid. Column was washed with 2 mL of 1 M formic acid, 2 mL of methanol, and 2 mL of 0.35 M NH_4_OH, applied in succession. Finally, cytokinins bases and ribosides were eluted with 2 mL of 0.35M NH_4_OH in 60% methanol (v/v). The eluted was evaporated to dryness in the RapidVap Evaporator and re-dissolved with 250 μL of methanol and 250 μL of 0.04% formic acid and centrifuged (20.000 × *g* and 10 min) before injection in HPLC-ESI-HRMS system. Description of the quantification and data processing was detailed in Silva-Navas et al. (2019).

### Root PM H^+^-ATPase Activity

Plasma membrane vesicles were extracted from the apical part of the roots (3–5 cm, 2 g (FW) from two plants per sample) using a sucrose-gradient technique as described in Mora et al. (2010). Extraction of vesicles (and subsequent enzymatic activity determination) was performed in quintuplicates (two plants per replicate) for each treatment and harvest time.

Briefly, apical roots were cut and ground in a mortar with a pestle in an ice cold extraction buffer containing: 250 mM sucrose, 10% (v/v) glycerol, 10 mM glycerol-1-phosphate, 2 mM MgSO_4_, 2 mM EDTA, 2 mM dithiothreitol (DTT), 2 mM EGTA, 2 mM ATP, 1 mM PMFS, 20 mg mL-1 chymostatin, 5.7% (w/v) choline-iodine, and 25 mM BTP (1,3-bis [TRIS (hydroxymethyl) methylamino] propane) buffered to pH 6.7 with MES. The homogenate mix was filtered through four layers of sterile gauze and then centrifuged 3 min at 13.000 × *g* and 4°C. The supernatant was conserved and centrifuged 25 min again under the same conditions. The pellets were recovered and resuspended in extraction buffer; this solution was loaded onto 1.5 mL tubes with the sucrose density gradient which consisted in 700 mL of 1.17 g/cm^3^ sucrose over 300 mL 1.13 g/cm^3^.

Sucrose solutions were prepared in 5 mM BTP-MES (pH 7.4) with all the protectants present in the extraction buffer. The gradients were centrifuged for 1 h at 13000 × *g*, and the vesicles banding at the interface were collected, resuspended again in extraction buffer for cleaning the residuals of sucrose, and centrifuged for 30 min at 13000 × *g*. The resulting pellets were resuspended in 0.5 mL of conservation buffer (20% glycerol; 5 mM DTT; 0.5mM ATP; 50 μg/ml chymostatin; 2 mM EDTA; 2mM EGTA; 2 mM BTP buffered with MES; pH 7.0). Finally, the PM vesicles were frozen with liquid N_2_ and stored at −80°C for enzyme activity measurements.

Enzyme activity was measured following the guidelines of ATPase/GTPase Assay Kit (DATG-200 kit, BioAssay Systems ATPase/GTPase – QuantiChromTM). Total protein quantification was based on the Bradford assay (Bradford, 1976).

### Statistical Analysis

Significant differences (*p* ≤ 0.05) among treatments were calculated by using one-way analysis of variance (ANOVA) and the LSD Fisher *post hoc* test. All statistical tests were performed using the statistical package Statistica 6.0 (StatSoft, Tulsa, OK, United States).

## Results

### Foliar-Applied SHA Led to Significant Shoot and Root Growth Increases but Did Not Induce Changes in Leaf Nutrient Concentrations

**FIGURE 1:**
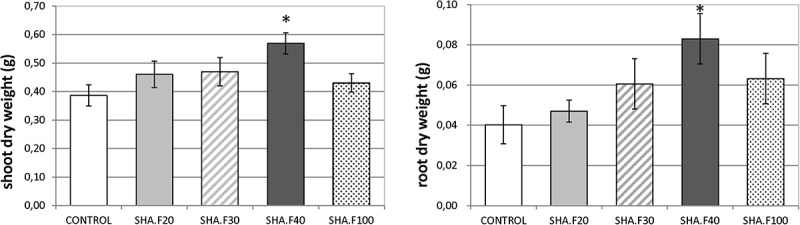
Effects of foliar application of different SHA doses (20, 30, 40, and 100 mg organic C L^–1^) on the shoot and root dry weight of cucumber plants after 72 h from the onset of treatments. The results are the mean ± *SE* (*n* = 5). Significant differences (Anova test; *p* ≤ 0.05) between treatments and control plants are indicated by an asterisk.

In the first set of experiments, we evaluated the dose-effect on plant growth. Leaves of cucumber plants were treated with four doses of SHA: 20, 30, 40 and 100 mg of organic C L^–1^ (SHA.F20, SHA.F30, SHA.F40, and SHA.F100). Seventy two hours from the onset of treatments, the only dose that showed significant increases in shoot and root dry matter was SHA.F40 (Figure 1). This dose (SHA.F40) was then selected for subsequent experiments. The foliar application of SHA did not cause any changes on the concentration of mineral nutrients in plant leaves (Supplementary Figure S3).

### Foliar-Applied SHA Led to Noticeable Changes in Root Architecture

**FIGURE 2:**
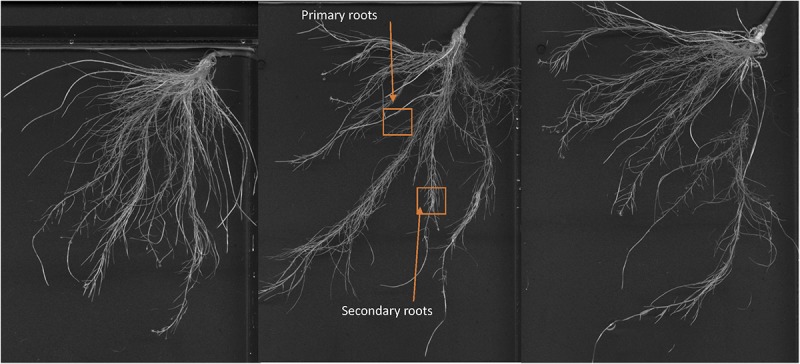
Whole root of cucumber control plants after 72 h from the onset of the treatments.

**FIGURE 3:**
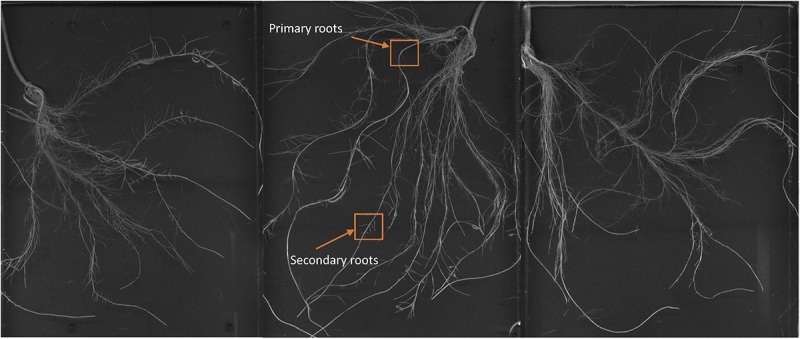
Whole root of cucumber plants 72 h after the foliar treatment with 40 mg C L^1^ of SHA (SHA.F40).

Images of the roots of cucumber plants corresponding to the control and foliar-applied SHA (SHA.F40) harvested 72 h from the onset of treatments are presented in Figures 2, 3. Noticeable effects on root architecture were observed upon SHA foliar-treatment. The qualitative analyses of the results indicated that the roots of control plants presented shorter principal roots but higher proportion of secondary roots than plants treated with SHA, which had longer principal roots but less density of secondary roots, as well as higher volume and more dry matter production (Figure 1).

### Foliar-Applied SHA Increased IAA but Decreased ABA, in Both the Root and the Shoot

**FIGURE 4:**
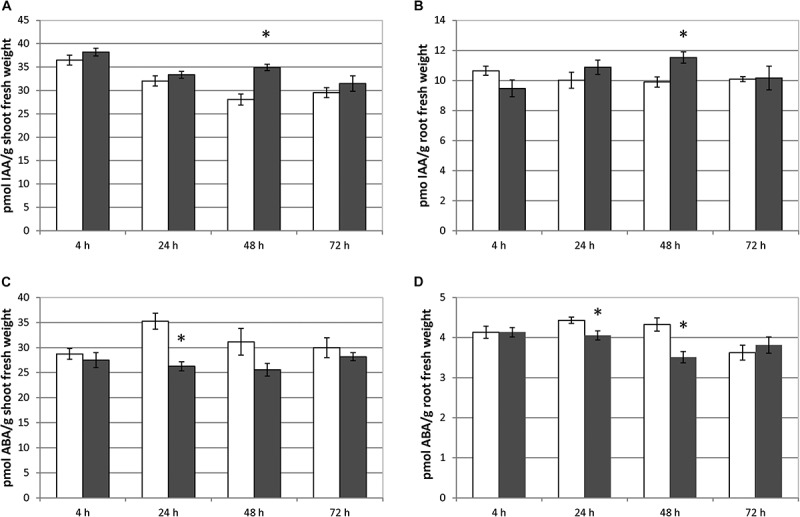
Effect of foliar applied SHA (40 mg C L^–1^, SHA.F40) on IAA concentration in shoots **(A)** and roots **(B)** and on ABA concentration in shoots **(C)** and roots **(D)** of cucumber plants (white bars: control; dark gray bars: SHA.F40). The results are the mean ± *SE* (*n* = 5). Significant differences (Anova test; *p* ≤ 0.05) between treatments and control plants are indicated by an asterisk.

Foliar-applied SHA.F40 caused a significant increase in IAA root concentration after 48 h from the onset of treatments (Figure 4A). This effect was accompanied by a concomitant increase in IAA concentration in the shoot also after 48 h from the treatment (Figure 4B). As for ABA, SHA.F40 decreased its concentration in both roots (after 48 and 72 h from the onset of treatment) and shoots (after 24 h from the onset of treatment) (Figures 4C,D).

### Foliar-Applied SHA Increased the Concentration of Several Cytokinins in Both Roots and Shoots

**FIGURE 5:**
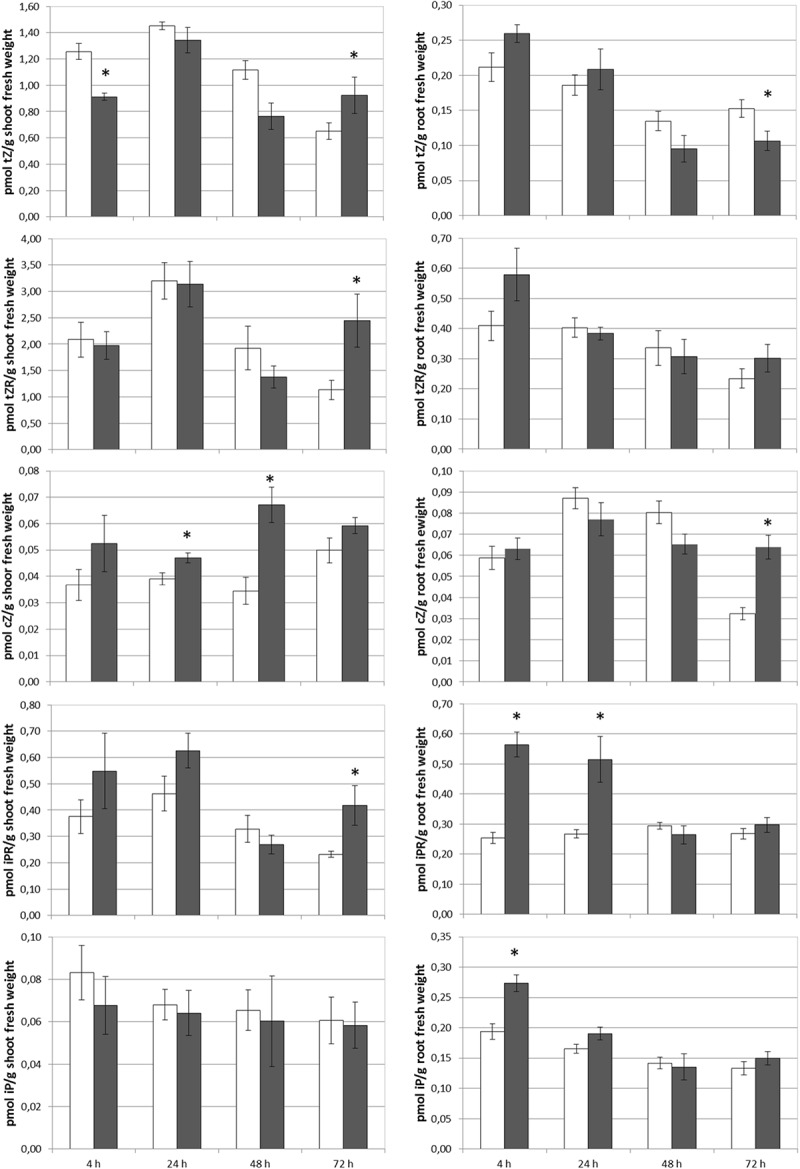
Effect of foliar applied SHA (40 gm C L^–1^, SHA.F40) on cytokinin concentration in shoots and roots (white bars: control; dark gray bars: SHA.F40). The results are the mean ± *SE* (*n* = 5). Significant differences (Anova test; *p* < 0.05) between treatments and control plants are indicated by an asterisk.

The foliar application of SHA.F40 caused an increase in the shoot concentrations of tZR after 72 h, cZ after 24 and 48 h, and iPR after 72 h (Figure 5). In the case of tZ a slight increase was observed after 72 h that was not significant (*p* = 0.13) (Figure 5). In the roots, SHA.F40 caused a significant increase in the concentration of iP after 4 h, iPR after 4 and 24 h, and cZ after 72 h (Figure 5). A slight increase in tZ after 4 h was also observed (*p* = 0.09) that was accompanied by a significant decrease after 72 h.

### Foliar-Applied SHA Did Not Induce Short-Term Increases in PM H^+^-ATPase- Activity in Plant Roots

**FIGURE 6:**
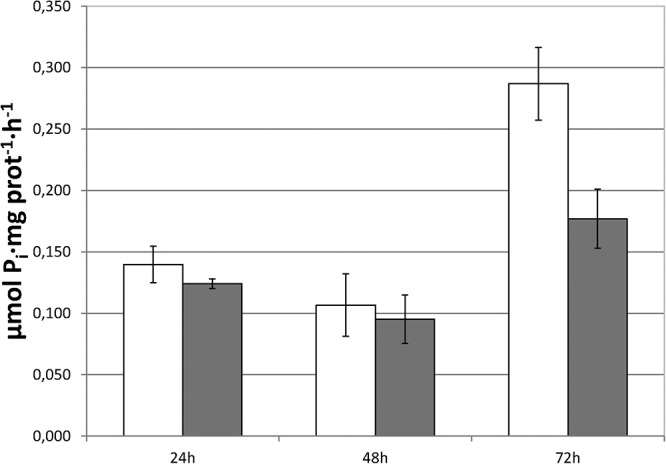
Root plasma membrane H^+^-ATPase activity of control and SHA foliar-treated plants. White bars: control; dark gray bars: 40 mg L^–1^ (SHA.40). The results are the mean ± *SE* (*n* = 5).

The capacity of foliar-applied SHA (SHA.F40) to increase the activity of root PM H^+^-ATPase activity was also studied. The results showed that SHA.F40 was not able to induce a short-term increase in the root PM H^+^-ATPase activity (Figure 6).

### Foliar-Applied SHA Led to Significant Increases in the Shoot- and Root- SA and JA/JAIle Concentrations

Considering that the deposition of SHA onto the leaves does not occur in nature and may present certain analogies with aggressions caused by external agents, the main plant hormones that are involved in the plant responses to this type of affection were also analyzed in roots and shoots: SA, JA, and JA-Ile.

**FIGURE 7:**
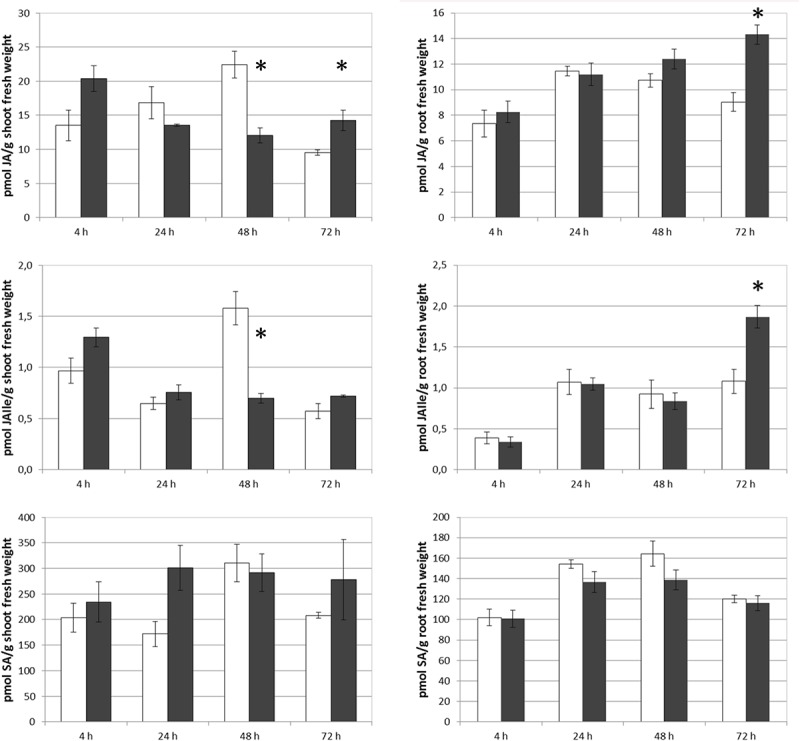
Effect of foliar applied SHA (40 gm C L^–1^, SHA.F40) on shoot and root concentration of JA, JAIle, and SA of cucumber plants (white bars: control; dark gray bars: SHA.F40). The results are the mean ± *SE* (*n* = 5). Significant differences (Anova test; *p* < 0.05) between treatments and control plants are indicated by an asterisk.

The results obtained show that SHA.F40 caused a significant increase in the root concentration of JA and JA-Ile after 72 h from the onset of treatments, whereas SA concentration was not affected (Figure 7). In shoots, however, SHA.F40 caused an increase in JA after 72 h and tended to increase SA concentration after 24 h (*p* = 0.081) and JA-Ile concentration after 4 h (*p* = 0.065) (Figure 7).

**FIGURE 8:**
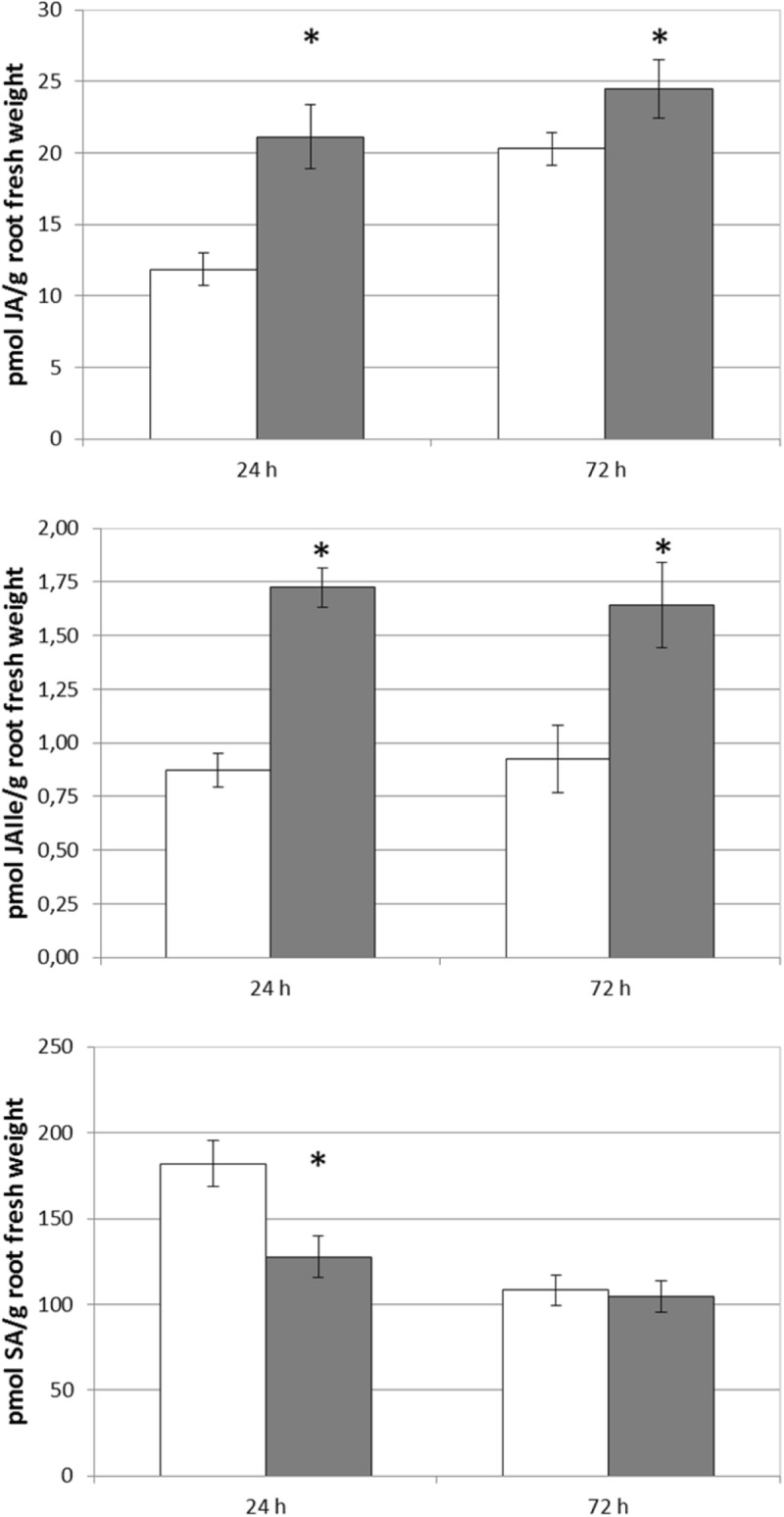
Effect of root applied SHA (100 gm C L^–1^, SHA.R100) on root concentration of JA, JAIle, and SA of cucumber plants (white bars: control; dark gray bars: SHA.R100). The results are the mean ± *SE* (*n* = 5). Significant differences (Anova test; *p* < 0.05) between treatments and control plants are indicated by an asterisk.

In order to compare these results with those corresponding to SHA-root application, and considering that there were no previous experimental results regarding the effects of root-applied SHA on the root and shoot concentration of SA, JA, and JA-Ile, the effect of 100 mg L^–1^ root-applied SHA (SHA.R100) on the concentration of these plant hormones was also investigated in cucumber. The results obtained show that SHA.R100 did not have a significant effect on the shoot-concentration of SA and JA/JA-Ile for the considered sampling times (data not shown), whereas a significant increase in both JA and JA-Ile was observed in the roots (Figure 8).

### Foliar-Applied SHA Affected Leaf Surface Structure and Mesophyll Cell Starch

**FIGURE 9:**
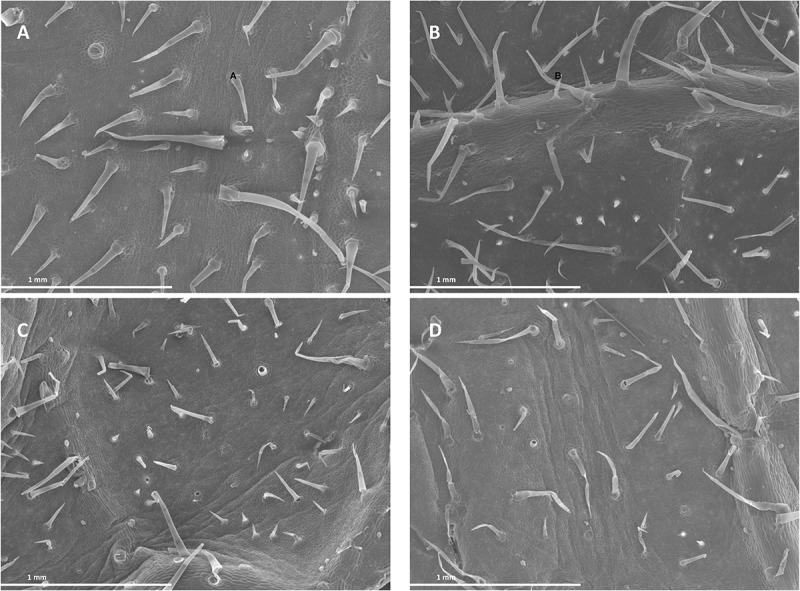
Scanning electron micrographs of cucumber leaf surfaces 7 days after foliar application: adaxial **(A)** and abaxial **(C)** leaf side of control leaves, adaxial **(B)** and abaxial **(D)** leaf side of 40 mg L^–1^ SHA-sprayed leaves (SHA.F40).

**FIGURE 10:**
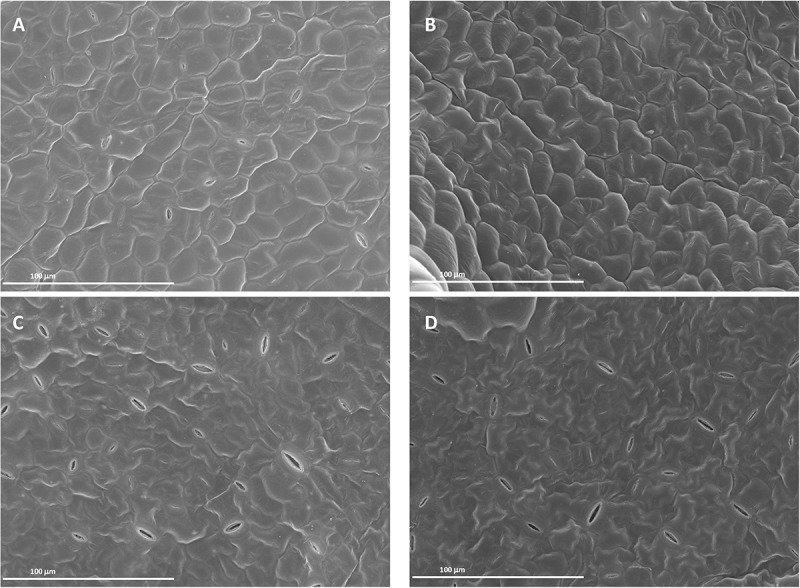
Scanning electron micrographs of cucumber leaf surfaces 7 days after foliar application: adaxial **(A)** and abaxial **(C)** leaf side of control leaves, adaxial **(B)** and abaxial **(D)** leaf side of 40 mg L^–1^ SHA-sprayed leaves (SHA.F40).

Images from both scanning (SEM) and transmission electron microscopy (TEM) revealed that the foliar application of SHA.F40 SHA affected some leaf structures, such as trichomes, cuticles, and starch granules. Images from SEM showed that the leaves of plants treated with foliar-applied SHA have undergone a loss of trichomes in both adaxial and abaxial leaf sides, compared to control plants (Figure 9), whereas there were no differences in the number of stomata or in the proportion of open/closed stomata (Figure 10). This result is in line with the values of stomatal conductance, which showed that there were no statistical differences between the stomatal conductance of control plants and SHA.F40 treated plants (data not shown).

**FIGURE 11:**
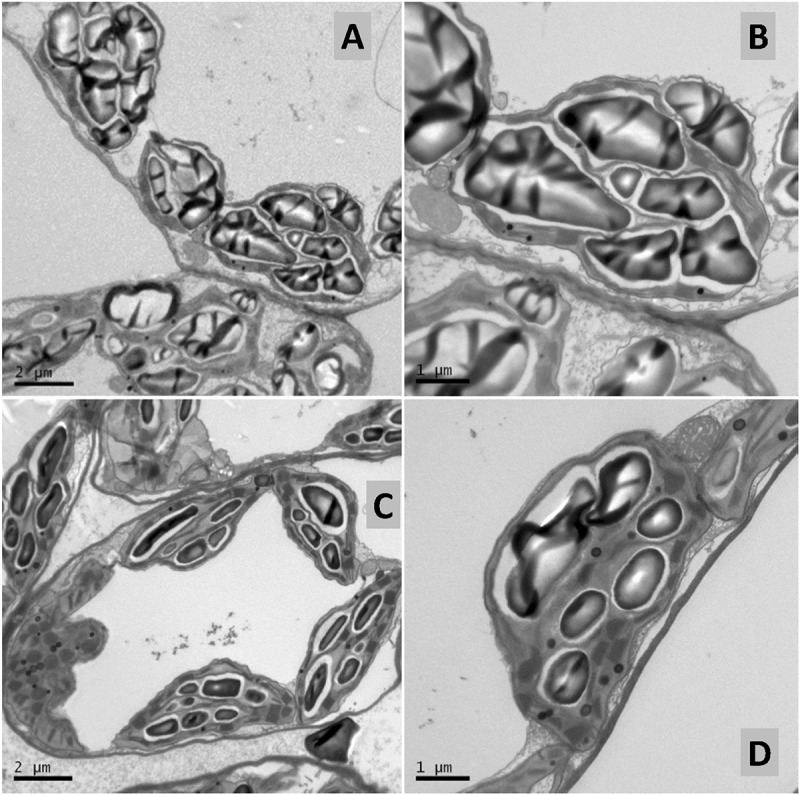
Transmission electron micrographs of control **(A,B)** and 40 mg L^–1^ SHA-sprayed (SHA.F40) **(C,D)** cucumber leaves, 7 days after foliar treatment. Detail of chloroplasts containing starch in mesophyll cells.

The foliar treatment with SHA.40 also caused a diminution of the size of starch granules present in the chloroplasts, in comparison with non-treated leaves from control plants (Figure 11).

## Discussion

### Different Mechanisms Underlay the Plant Growth Promoting Action of Foliar- Versus Root-Applied HA

In agreement with previous results on the application of HS to plant leaves (Rose et al., 2014; Canellas et al., 2015), foliar-applied SHA was found to promote significant increases in both shoot and root dry matter at the concentration of 40 mg C L^–1^ (SHA.F40) (Figure 1). These results are in line with the results obtained with root-applied SHA in cucumber plants cultivated in hydroponics under the same environmental and nutritional conditions as that used in the present study (Aguirre et al., 2009; Mora et al., 2010, 2012, 2014; Olaetxea et al., 2015, 2019). In principle, these results indicate that SHA is able to promote plant growth regardless the mode of application. However, this fact does not mean that the mechanisms of action underlying these effects are similar to each other.

In fact, some differences were observed regarding the effects on root morphology and architecture. Many studies have reported the capacity of root-applied HA to promote the proliferation of secondary roots (Nardi et al., 2002; Zandonadi et al., 2010; Canellas et al., 2012; García et al., 2016a; Olaetxea et al., 2018). In the case of other studies involving cucumber plants cultivated in hydroponics under similar conditions as in the present study, Mora et al. (2012) reported that root-applied SHA promoted the number of secondary roots as well as root growth in short-term experiments. However, the short-term response to foliar-applied SHA showed that SHA tended to reduce the presence of secondary roots with respect to control plants and increase principal root length and root dry weight with respect to the control (Figures 1–3). This fact might be related to the different effect of foliar-applied SHA and root-applied SHA on the concentration in roots of two phytoregulators related to the regulation of root growth and architecture: IAA and ABA. Several studies have shown that the capacity of root-applied HA to enhance lateral root proliferation appears to be mediated by auxin and nitric oxide signaling pathways (Nardi et al., 2002; Zandonadi et al., 2010; Canellas et al., 2015; Olaetxea et al., 2018). Other studies in cucumber with a similar experimental design and conditions as reported here showed that SHA applied to the roots increased the root concentration of IAA and ABA (Mora et al., 2012; Olaetxea et al., 2015). However, whereas inhibitors of IAA biosynthesis and action affected secondary root development but not the SHA-mediated increase in root dry matter (Mora et al., 2012), the inhibition of ABA biosynthesis prevented the SHA effect on the whole root growth reflected in root dry matter production (Olaetxea et al., 2019). These results suggested a relevant role of ABA in the mechanisms underlying the action of root-applied SHA on the root development (Olaetxea et al., 2019). However, the results obtained in our experiments indicate that some other factor (or factors) must be affecting the effect of foliar-applied SHA on root growth and architecture. This conclusion is supported by the fact that we do not observe any increase in secondary root proliferation linked to the increase in root IAA, but we do observe an increase in the global root growth as reflected by the dry matter production despite root ABA concentration did not increase. As it will be discussed later, these results might be associated with the crosstalk between the different hormones affected by foliar-applied SHA, rather than with an effect of a specific hormone. In any case, it is clear that the short-term effects of SHA applied to the leaves and those for SHA applied to the roots show different patterns regarding root development and architecture.

Previous studies reported that the improvement in cucumber shoot growth associated with root-applied SHA was linked to the increase in IAA caused by SHA in the roots (Mora et al., 2014). SHA applied to the leaves also caused an increase in the concentration of IAA in roots (Figures 4A,B). Interestingly, this effect was accompanied with an increase of IAA concentration in the shoot (Figures 4A,B), which might play a relevant role in the promotion of shoot growth associated with foliar-applied SHA since several studies have reported its role in the regulation of stem elongation and shoot growth (Gallavotti, 2013). In summary, these results support that IAA could also play a relevant role in the shoot growth promotion resulting from foliar-applied SHA.

The decrease in ABA in root and shoot linked to the foliar application of SHA may be relevant regarding shoot growth. It is well known that increases in ABA in the shoot are normally associated with a decrease in shoot growth (Vysotskaya et al., 2018) and leaf senescence promotion (Ghanem et al., 2008). It is therefore possible that the decrease in shoot ABA caused by foliar-applied SHA might have also contributed to the shoot growth.

Further studies in cucumber showed that root PM H^+^-ATPase activity played a crucial role in the shoot growth-promoting action of root-applied SHA (Olaetxea et al., 2019). In fact, the use of inhibitors of the activity of this enzyme prevented the increase in shoot growth mediated by SHA applied to roots (Olaetxea et al., 2019). It is therefore plausible that this enzyme may also be involved in the increase in shoot growth caused by foliar-applied SHA. However, the results obtained in experiments with foliar-applied SHA.F40 associated with short-term increases in shoot growth, did not show any noticeable short-term effect on root PM-H^+^-ATPase activity (Figure 6). Therefore, although a medium- and/or long- term stimulation of root PM H^+^-ATPase activity resulting from foliar-applied SHA cannot be ruled out, this action would not explain the short-term enhancement of shoot growth promoted by foliar SHA application (Figure 1). In addition, the lack of effects of foliar-applied SHA on the root PM H^+^-ATPase activity may explain why foliar applied-SHA did not change the leaf concentration of the nutrients analyzed (Supplementary Figure S3) since this enzyme is directly involved in root nutrient uptake (Olaetxea et al., 2018).

Another event that played a relevant role in the mechanism underlying the shoot-growth promoting action of root-applied SHA was a short-term increase in the concentration of some cytokinins in the leaves and roots (Mora et al., 2010). In the case of foliar-applied SHA we also observed an increase in the root and shoot concentration of several cytokinins (Figure 5). This fact is in line with the enhancement of shoot growth observed in foliar-SHA treated plants. In the case of root-applied SHA the effect of cytokinin concentration in leaves was mediated by the stimulation in root-PM H^+^-ATPase activity (Olaetxea et al., 2019). Nevertheless, for foliar-applied SHA this mechanism does not appear to be involved in the regulation of this process since this treatment did not have any short-term effect on root-PM H^+^-ATPase activity.

Olaetxea et al. (2015, 2019) reported that root ABA also played an important role in the promotion of shoot growth after root SHA application (Olaetxea et al., 2015, 2019). However, foliar-applied SHA did not increase ABA concentrations in roots (Figure 4), thus suggesting that this event is not involved in its effect on shoot growth. Therefore, in addition to IAA, other signaling pathways different from root PM- H^+^-ATPase and root ABA must be involved in the shoot growth promoting action of foliar-applied SHA and the increase in cytokinin leaf concentration resulting from this treatment.

### SHA Applied on the Leaves, but Also to the Roots, Affects SA and JA Signaling Pathways

As described in the introduction, the interaction of HS with leaf surfaces does not occur in nature and can be sensed by plants as an external aggression. In such case, plants normally activate SA and JA/JA-Ile signaling pathways as a defensive and adaptive response (Wasternack and Hause, 2013; Nazar et al., 2017). It is therefore plausible that foliar-applied SHA may activate these signaling pathways. In this framework, the results obtained regarding the root- and shoot- concentration of SA and JA/JA-Ile are very relevant. Our results confirm this hypothesis since SHA applied to leaves clearly affected the concentration in roots and shoots of JA and JA-Ile that is the active form of the hormone (Figure 7).

These results suggest that foliar-applied SHA may cause some damage at a leaf surface level. Analysis of foliar-SHA treated leaves by SEM and TEM showed some anatomical changes associated with SHA application.

On the one hand, SHA treatment decreased trichome densities (Figure 9). A further interesting finding was the decrease in leaf mesophyll starch accumulation in the chloroplasts upon SHA foliar application (Figure 11). This effect was unexpected since the application of HA to plant roots is associated with an increase in chloroplast starch accumulation (Jannin et al., 2012). This effect may be potentially linked to a mobilization of carbohydrates associated with higher metabolic activity and regulated by cytokinin activity. However, the effect of foliar SHA supply of leaf starch concentrations should be studied more in depth in future investigations.

In order to compare the effects of SHA foliar application on JA, JA-Ile, and SA with those obtained with root-applied SHA, we carried out a new experiment exploring the action of SHA applied to the roots on the concentration of these hormones in roots and shoots. This experiment was performed in cucumber plants cultivated in hydroponics under the same environmental and nutritional conditions as that used in foliar SHA application and preliminary root SHA supply trials (Olaetxea et al., 2019). Surprisingly, SHA root application led to significant short-term increases in the root concentration of both JA and JA-Ile (Figure 8), whereas no clear effects were observed in shoots. As in the case of foliar-applied SHA, these results are consistent with some potential involvement of JA signaling pathway in the whole mechanism of action of root-applied SHA on plant growth.

Regarding the potential roles that SA and JA could play in the mechanisms responsible for the plant growth-promoting action of SHA applied to either roots or leaves, several studies reported negative cross-talk between SA and JA in the regulation of several processes related to plant development, such as plant defense mechanisms and root development (Traw and Bergelson, 2003). Likewise, it is well known that SA is generally involved in the regulation of plant responses to biotrophic and hemi-biotrophic pathogens, whereas JA is involved in plant responses to necrotrophic pathogens and herbivorous (Wasternack and Hause, 2013; Nazar et al., 2017). In this context, it is therefore complicated to discuss the role of both SA and JA in the positive regulation of the same process.

Some studies described that the application of low concentrations of SA increased root growth and root dry matter production (Deef, 2007). Conversely, several studies reported that JA inhibited plant growth but promoted secondary root formation (Wasternack and Hause, 2013). In our experiments with foliar-applied SHA, we observed short-term increases in JA and JA-Ile root concentrations that were not accompanied by a reduction in root growth or increases in lateral root formation (Figures 9A, 3, 4, respectively). On the contrary, we observed an increase in root dry matter production and a reduction in secondary root formation (Figures 3, 4). This fact suggests that JA signaling pathways do not play a dominant role in short-term effects of foliar-applied SHA on root development. As mentioned above, these results suggest that these processes might be regulated by the ratios, the relative proportion, between specific hormones involved in root development regulation such as IAA, ABA, cytokinins, SA and JA.

However, regarding root-applied SHA, the results obtained are compatible with a relevant role of JA in the SHA mediated effects on secondary root production along with other hormones such as IAA and ABA (Olaetxea et al., 2015, 2019).

Finally, the effects of foliar-applied SHA on JA signaling pathways are compatible with the induction of higher resistance of treated plants against eventual pathogen attacks. In any case, it becomes clear that more research is required in order to elucidate the role of JA in the whole mechanism underlying the beneficial action of SHA on plant development and, eventually, plant defense against pathogens.

Likewise, it is highly likely that additional biochemical and molecular processes may also be involved in the long-term response of plants sprayed with HS. However, in light of our findings, the short-term reaction of plants to HS application has great influence in the whole action of HS during the entire growing cycle (Olaetxea et al., 2018).

## Conclusion

**FIGURE 12:**
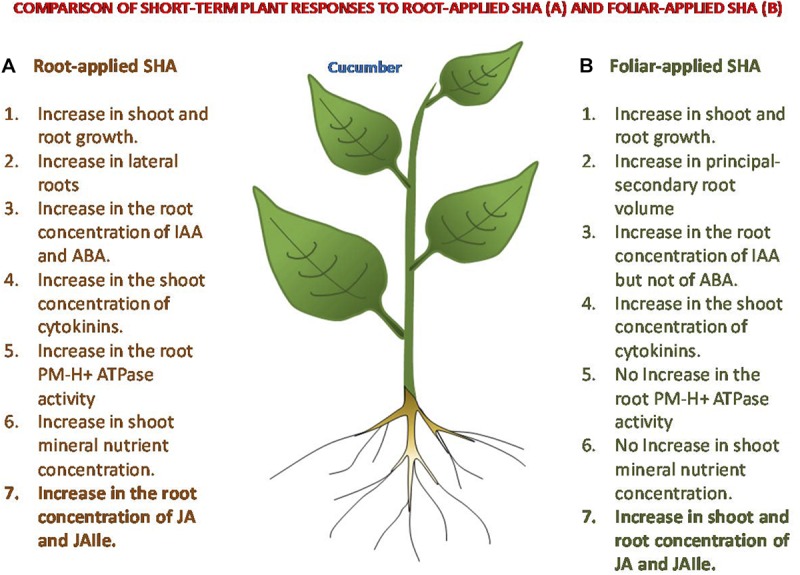
Comparison of some short-term responses on cucumber plants to root-applied SHA and foliar-applied SHA.

The results obtained are compatible with the hypothesis that the beneficial action of foliar-applied SHA or root-applied SHA on plant growth may result from molecular and biochemical events triggered by a transient mild stress associated with SHA application (Figure 12), although the mechanisms underlying these responses are different depending on the mode of application. Whereas the root application of SHA increases plasma membrane H^+^-ATPase activity, shoot mineral nutrient concentration, and ABA concentrations in roots, among other effects, foliar-applied SHA did not induce those effects. However, both root-applied and foliar-applied SHA caused increases in IAA cytokinins, JA and JA-Ile. In this sense, further studies are needed in order to unveil the role of JA in the mechanisms of action of SHA.

## Data Availability Statement

All datasets generated in this study are included in the article/Supplementary Material.

## Author Contributions

DD, MF, VF, and JG-M conceptualized and designed the study. DD, MF, and MO performed the experimental work. AZ assessed the hormone detection. VF performed the microscopy studies. DD, MF, and JG-M analyzed the data. JG-M, DD, MF, VF, and AZ prepared the manuscript.

## Conflict of Interest

The authors declare that the research was conducted in the absence of any commercial or financial relationships that could be construed as a potential conflict of interest.
