# Supplementary material for: Discriminating the Short-Term Action of Root and Foliar Application of Humic Acids on Plant Growth: Emerging Role of Jasmonic Acid
Source: Front Plant Sci. 2020 Apr 28;11:493. doi: 10.3389/fpls.2020.00493 (PMC7199506; doi:10.3389/fpls.2020.00493)
Supplement: Supplementary file 1 [file Image_1.pdf]

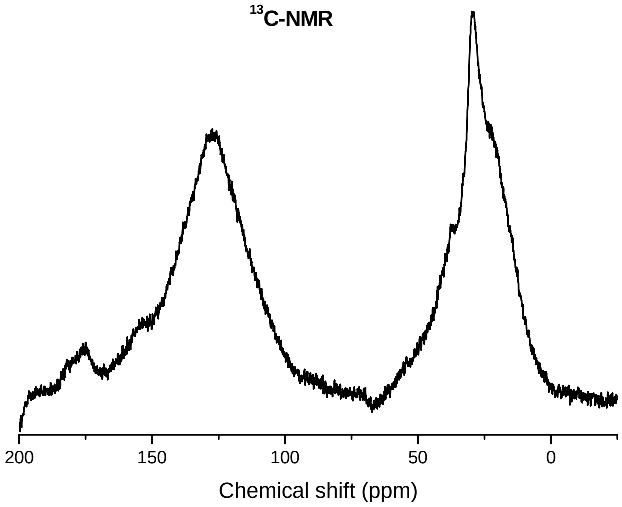

Region (ppm)	Alkyl C 0–45	O-alkyl C 45–110	Aromatic C (Phenolic C) 110–160 (140–160)	Carboxylic C 160–190	Carbonylic C 190–230
SHA (%C)	31.4	14.4	38.6 (14.7)	11.6	3.90

Figure. S1. ^{13}C-NMR for SHA
