# Supplementary figures and images for: Discriminating the Short-Term Action of Root and Foliar Application of Humic Acids on Plant Growth: Emerging Role of Jasmonic Acid
Source: Front Plant Sci. 2020 Apr 28;11:493. doi: 10.3389/fpls.2020.00493 (PMC7199506; doi:10.3389/fpls.2020.00493)

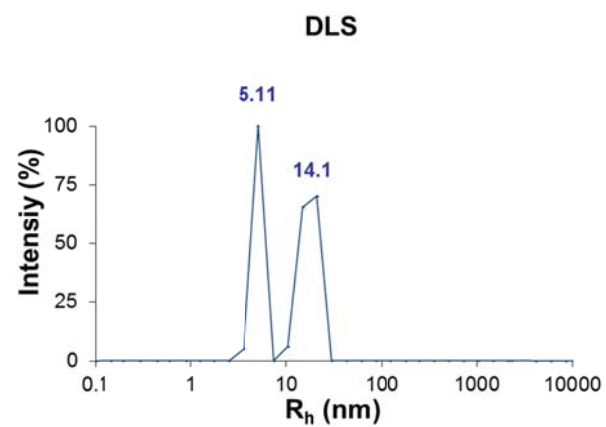

Figure S2. Dynamic light scattering (DLS) analysis of size SHA populations in solution.

Supplement: Supplementary file 2 [file Image_2.pdf]

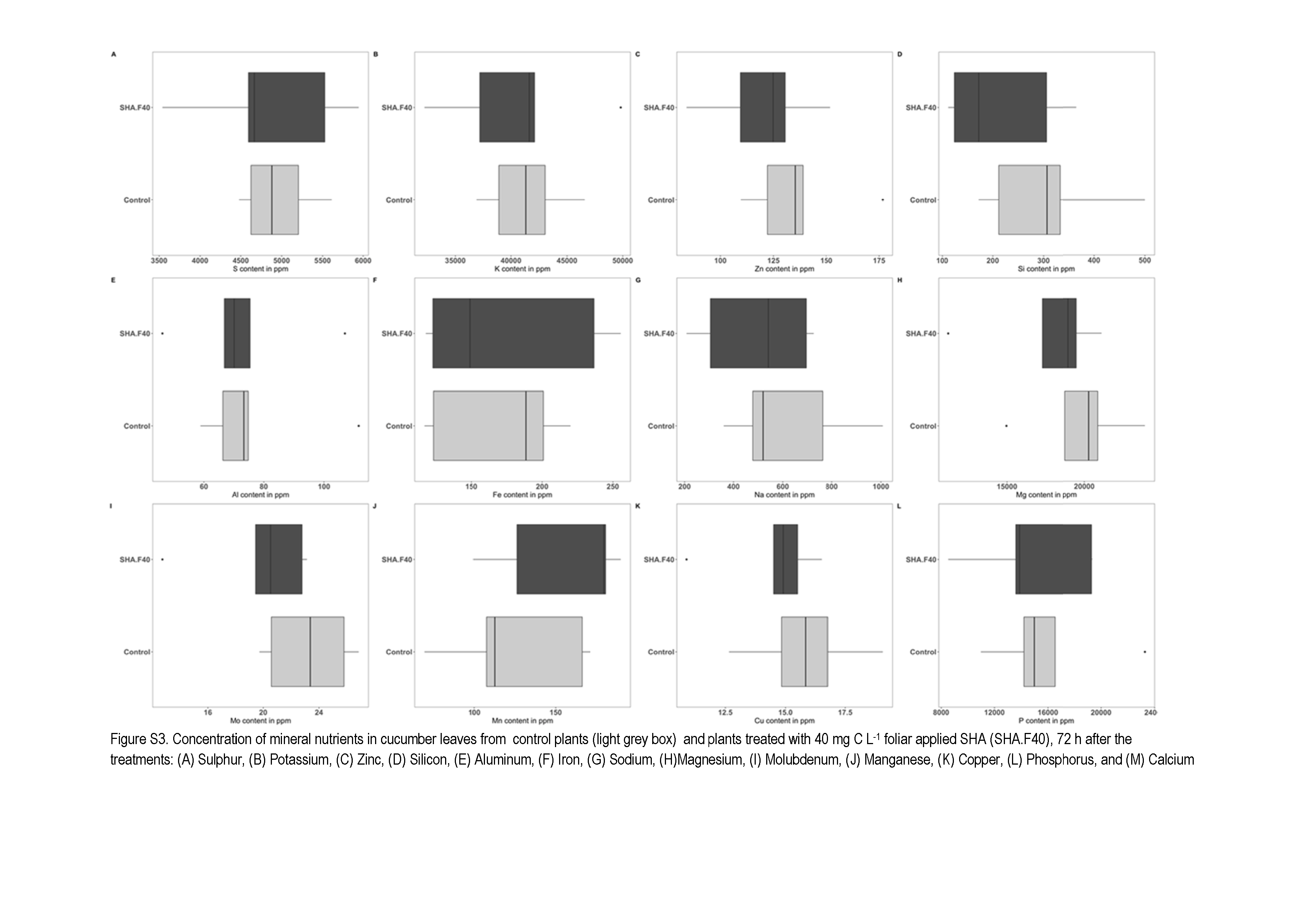

Supplement: Supplementary file 3 [file Image_3.tif]
