# Supplementary material for: Discriminating the Short-Term Action of Root and Foliar Application of Humic Acids on Plant Growth: Emerging Role of Jasmonic Acid
Source: Front Plant Sci. 2020 Apr 28;11:493. doi: 10.3389/fpls.2020.00493 (PMC7199506; doi:10.3389/fpls.2020.00493)
Supplement: Supplementary file 4 [file Table_1.DOCX]

Table S1. Elemental Analysis of SHA

|  | **%C** | **%H** | **%N** | **%S** | **%O^*^** |
| --- | --- | --- | --- | --- | --- |
| SHA | 48.2 | 2.99 | 0.98 | 0.14 | 47.7 |
|  |  |  |  |  |  |
| ^*^ by difference | |  |  |  |  |
